# EXPLORING THE EFFECTIVENESS OF COGNITIVE STIMULATION THERAPY FOR DEMENTIA IN UNITED STATES, BRAZIL, AND HONG KONG

**DOI:** 10.1093/geroni/igae098.4185

**Published:** 2024-12-31

**Authors:** Ebow Nketsiah

**Affiliations:** Saint Louis University, St. Louis, Missouri, United States

## Abstract

Cognitive Stimulation Therapy (CST) is a cost-effective non-pharmacological intervention for dementia developed in the United Kingdom. Though CST has been adopted and implemented all over the world, there has not been a study to evaluate its effectiveness across multiple counties. This study analyzes existing data from CST groups in USA, Hong Kong, and Brazil to evaluate the primary outcomes CST for persons with dementia. Paired sample t-tests were conducted to evaluate the effect of CST on cognition, quality of life, physical function, and depression across the countries. Among the USA CST groups (N=288), significant t-test were calculated for cognition, t(261) = -7.412, p< 0.01, quality of life, t(199) = -8.707, p< 0.01, and depression, t(254) = 6.347, p< 0.01. A non-significant t-test was calculated for physical function, t(103) = 1.262, p=0.21. Among the Hong Kong CST groups (N=408), significant t-tests were calculated for cognition, t(204) = 3.125, p< 0.01, depression, t(236) = 3.084, p< 0.01. However, non-significant t-tests were calculated for quality of life, t(376) = -0.614, p=0.539, physical function, t(84) = -0.888, p=0.377. On the other hand, among Brazil CST groups (N=47), significant t-test was calculated for cognition, t(46) = -2.961, p< 0.01. However, non-significant t-tests were calculated for quality of life, t(46) = -1.760, p=0.085, physical function, t(46) = -0.250, p=0.804, and depression, t(46) = 0.345, p=0.690. There are cultural and demographic factors that influence the effectiveness of interventions like CST. As CST is being implemented as a cost-effective non-pharmacological intervention for dementia and Alzheimer’s worldwide, its cultural applicability is worth exploring.

